# Age-related differences and sexual dimorphism in canine sleep spindles

**DOI:** 10.1038/s41598-019-46434-y

**Published:** 2019-07-12

**Authors:** Ivaylo Borislavov Iotchev, Anna Kis, Borbála Turcsán, Daniel Rodrigo Tejeda Fernández de Lara, Vivien Reicher, Enikő Kubinyi

**Affiliations:** 1 Department of Ethology, Eötvös Loránd University, Budapest, Hungary; 2 Institute of Cognitive Neuroscience and Psychology, Hungarian Academy of Sciences, Budapest, Hungary; 3 Faculty of Veterinary Medicine and Animal Husbandry, National Autonomous University of Mexico, Mexico City, Mexico

**Keywords:** Cognitive ageing, Neurophysiology

## Abstract

Non-REM bursts of activity in the sigma range (9–16 Hz) typical of sleep spindles predict learning in dogs, similar to humans and rats. Little is known, however, about the age-related changes in amplitude, density (spindles/minute) and frequency (waves/second) of canine spindles. We investigated a large sample (N = 155) of intact and neutered pet dogs of both sexes, varying in breed and age, searching for spindles in segments of non-REM sleep. We recorded EEG from both a frontal midline electrode (Fz) and a central midline electrode (Cz) in 55.5% of the dogs, in the remaining animals only the Fz electrode was active (bipolar derivation). A similar topography was observed for fast (≥13 Hz) spindle occurrence as in humans (fast spindle number, density on Cz > Fz). For fast spindles, density was higher in females, and increased with age. These effects were more pronounced among intact animals and on Fz. Slow spindle density declined and fast spindle frequency increased with age on Cz, while on Fz age-related amplitude decline was observed. The frequency of fast spindles on Fz and slow spindles on Cz was linked to both sex and neutering, suggesting modulation by sexual hormones. Intact females displayed higher frequencies than males and neutered females. Our findings support the argument that sigma bursts in the canine non-REM sleep are analogous to human sleep spindles, and suggest that slow and fast spindles display different trajectories related to age, of which an increase in frontal fast spindles is unique to dogs.

## Introduction

The sleeping brain shows various patterns of activity that predict awake performance in the domains of memory and cognitive ability^1^. Several of these oscillatory activities are also altered in the aging process^2^, which makes sleep physiology an attractive target for studying cognitive aging in humans and animal models. The dog has been suggested to be a suitable model for human cognitive aging^3–5^. Their advantage over rodents in this respect is argued on the basis of a more similar behavioural repertoire^6^ and a shared environment^7^. Recently sleep physiology in dogs has received growing attention^8–11^ due to the development of a non-invasive polysomnographic method^8^ which opens up a possibility for integrating the study of cognitive aging and sleep in pet dogs.

The sleep spindle, which appears as a short (0.5–5 seconds) train of rhythmic and symmetric waves^12,13^, traceable with EEG and occurring predominantly during non-REM sleep, had been described, but seldom quantified in the dog. Various contradicting accounts about its defining features in canines had been published based on visual inspection alone^8,14–16^, derived from both invasive and non-invasive work. Using criteria for automatic sleep spindle detection previously validated in humans^17^ we were able to show that transients in the sigma range (9–16 Hz), characteristic of the human sleep spindle, show a similar association with post-sleep recall of novel information, i.e. dogs with higher learning gain displayed more spindles/minute^18^.

The sleep spindle is a particularly attractive target of investigation with regard to aging. Its rate of occurrence (density), amplitude, frequency (waves/second of a single spindle) and duration have each been found to change in humans, from childhood to puberty^19,20^, from young adult to old age^21–23^, and between healthy and pathologically aging groups^24–27^. Age-related changes, in particular those associated with cognitive decline, are characterized by decreased density (spindles/minute) and amplitude^2,23,24,26,27^. A subset of studies also found an increase in spindle frequency for older subjects^2^, whereas adolescent development is characterized by increased density^19,20^.

A distinction between fast and slow spindles is common in humans^12,13,28^. The two sub-types can be topographically distinguished. Fast spindles are predominantly found in central and posterior derivations^29–31^ and oscillate on average ≥13 Hz, whereas slow spindles (≤13 Hz) are predominant in the frontal derivations. The distinction is, however, less clear based on the origin of spindles. Invasive work in cats had originally implied a universal thalamic origin of spindles^32–34^. But although there is evidence for thalamic involvement in the generation of both types^35^ differences in pharmacological responsiveness have raised the question if they are equally controlled by the thalamus^36,37^. Importantly, in humans there is variation with regard to how sensitive spindle-associated findings are for this slow versus fast distinction. A positive correlation between spindle occurrence and memory performance, for instance, can be observed with^38,39^ and without^40–42^ separating spindles into ‘slow’ and ‘fast’, whereas many findings on development^19,20^ and sexual dimorphism^43^ are specific for the different subtypes, e.g. a rise in spindle density during adolescence appears to be specific for fast spindles^19^.

In addition to being potentially useful predictors of healthy aging, spindles have also received attention for being sexually dimorph^20–22,43–46^. The issue of which sex displays a higher rate of spindle occurrence is unsettled, but our previous observations in the dog^18^ square with findings suggesting higher spindle density in women^44^, but see^43,45^ reporting the opposite effect. In humans, measuring sexual differences in spindle density may be complicated by topographic differences in occurrence, i.e. for women a higher proportion of spindles appear over the left frontal cortex^22^ and interaction with medical conditions^21^ (i.e. spindle density seems to be elevated in women, but not men, suffering from depression). Generally spindle density in women varies in the course of the menstrual cycle, with the highest occurrence observed in the luteal phase, characterized by higher levels of progesterone^46^. A more reliable finding is that women have higher fast spindle amplitudes^43,45^, especially since invasive human recordings (in epilepsy patients and volunteers)^45^ exclude that sex differences in skull thickness account for the observed differences in amplitude. At least some sexually dimorph features of spindles, like fast spindle frequency, have been linked more directly to sexual hormones like progesterone and oestrogen^45^, therefore we can expect neutering to also affect spindle characteristics.

In the present investigation, we aim to compare spindle features across neutered and intact dogs from both sexes varying in age, relying on a large sample (>150 dogs) of subjects, which underwent ca. 3 hours of polysomnographic recordings with no additional experimental manipulation. Currently the literature on canine spindles is sparse and little is known about the development of spindling in dogs across their life span. Early work by Pampiglione^47^ is quoted to conclude that spindles are rare in young dogs (see Jankel and Niedermeyer^13^). Other works that mentions spindles in the dog did not quantify the events^16,48^. A notable exception in terms of developmental insight is the comparative work of Petersen *et al*.^49^ which showed a later postnatal expression of sleep spindles in dogs, compared to cats and rabbits (in dogs the first spindles appeared about a month after birth, while in rabbits between one and two weeks, and in cats two weeks to a month). Even this study, however, does not quantify spindles, but merely compare the time between species they become visually detectable.

In the absence of an equally well-developed body of findings in the dog, yet emerging evidence for an analogy between human and canine spindles^18^, we base our expectations on the human literature. It should be noted, however, that the most confirmed analogy between humans and other mammals to date remains the association between spindle expression and learning^18,50,51^. As far as dog-specific findings are concerned, the literature only contains information on the development of sleep spindles in young dogs compared to the young of other species. Sleep spindles appear later in dogs compared to rabbits and cats^49^, but not humans^52^. Pampiglione^53^ suggested that dog brains generally mature slower in comparison with pigs. A previously observed increase in frontal spindles with age in the dog^18^ fits with this late onset of sleep spindles, but the change in spindle occurrence across a dog’s lifespan remains an open question, to be investigated in the present study. In humans age-related increases in spindle occurrence last only until adolescence^19,20^. A lifelong increase in spindle density does therefore not seem likely, especially since in humans a decrease is observed among the eldest^2,23,24,26,27^. We further expect to find analogies to humans with regard to sex differences in spindle density, amplitude and frequency^22,43–45^, since universally mammalian sexual hormones like progesterone and oestrogen are found to modulate spindle features in humans^45,46^. Among the three spindle features we expect amplitude-related findings to be most robust, as a recent meta-analysis suggests that findings associated with spindle amplitude are the overall most reliable^54^.

## Methods

### Ethics statement

Research was carried out in accordance with the Hungarian regulations on animal experimentation and the Guidelines for the use of animals in research described by the Association for the Study Animal Behaviour (ASAB). The Hungarian “Animal Experiments Scientific and Ethical Committee” issued a statement (under the number PE/EA/853–2/2016), approving our experimental protocol by categorizing it as a non-invasive study that causes less pain or suffering than the equivalent of inserting a needle. All owners volunteered to participate in the study.

### Subjects

155 dogs (age range 1–16 years, 7.6 ± 4 (M ± SD); 76 females; 107 neutered and 11 of unknown reproductive status; 96 purebred from 39 different breeds) were taken from our Family Dog Project database consisting of ca. 3 hour long, first-time polysomnographic recordings with no additional experimental manipulations. Dogs that did not sleep during the recording (N = 8 in the full sample, N = 2 in the subsample with an active Cz electrode) were excluded from all analyses, while dogs that slept but did not express spindles (N = 1 in the full sample, none in the subsample with active Cz electrode) were also excluded from analyses of amplitude and frequency. For analyses focusing on fast spindles (≥13 Hz) more dogs were excluded from amplitude and frequency comparisons (additional N = 20 in the full sample, 7 in the subsample with an active Cz electrode) due to a higher proportion of dogs displaying no fast spindles. One additional dog was excluded in the amplitude and frequency analyses for slow spindles, as it only showed detections ≥13 Hz.

### Polysomnographic method

A detailed description of the polysomnographic method can be found in Kis *et al*.^8,9^. The data was gathered in 2012–2019. During this period the recording methods and electrode placements were upgraded. In all dogs, electrodes were placed on the skull midline (Fz, Cz, Pz). The anterior midline electrode (Fz) was active in all dogs, but Cz was active in only 86 animals (55.5% of the total sample). Both Fz and Cz (where applicable) was referred to Pz, placed on the occipital bone at the back of the dog’s head. The remaining head electrodes consisted of a ground electrode at the left musculus temporalis and one or two additional electrodes for measuring eye movements (placed on the left and right os zygomioticum). Furthermore Electrocardiogram (ECG), respiration and muscle tone was monitored in order to aid sleep stage identification. The impedance of the active electrodes was kept below 20 kΩ. Furthermore, recordings in our database were obtained with one of the following two technical arrangements:

1. In 27 dogs (17.4% of the total sample) the signal was collected, pre-filtered, amplified and digitalized with a sampling rate of 249 Hz/channel using a 30-channel Flat Style SLEEP La Mont Headbox with implemented second order filters (high pass >0.5 Hz, low pass <70 Hz), and HBX32-SLP 32 channel pre-amplifier (La Mont Medical Inc., USA).
2. In 128 dogs (82.6% of the total sample) the signal was collected, pre-filtered, amplified and digitized with a sampling rate of 1024 Hz/channel using a SAM 25 R style MicroMed Headbox (MicroMed Inc., Houston, TX, USA). The hardware passband was set at 0.5–256 Hz, sampling rate of 512 Hz, anti-aliasing filter with cut-off frequency at 1 kHz, and 12-bit resolution covering a voltage range of ±2 mV as well as second-order software filters (high pass >0.016 Hz, low pass <70 Hz) using System Plus Evolution software (MicroMed Inc, Houston, TX, USA).

To account for the use of different recording set-ups, we had to switch from a discrete time zero-pole-gain representation to a second-order section representation of the Butterworth filter for offline filtering, while keeping the same attenuation coefficients, pass band and stop band cut-off frequencies, as previously described^18^. Second-order section representations of filters are based on equations that provide transfer-functions less sensitive for individual deviations in how different signal components are amplified or attenuated during sampling with different settings. As an additional control against systematic differences due to recording method, spindle measures were compared between the two groups in the Supplementary. Prior to filtering, the EEG signal was divided in sleep-stages using visual inspection, as in Kis *et al*.^9^ with the EEG viewing program *Fercio’s EEG Plus*.

### Spindle detection

Our detection script was previously described in Iotchev *et al*.^18^ and is closely modelled on search criteria validated in human children by Nonclercq *et al*.^17^. Another detailed description is also provided in the Supplementary material. Importantly, our algorithm uses normal modelling, i.e. potential detections above or below more than 2 standard deviations from the mean of all putative spindling events regarding amplitude or frequency were removed as outliers. To this end, the maximum likelihood estimates of the amplitude and frequency means and standard deviations were calculated for all initial detections per dog and recording. We use a relative measure of amplitude (distance from the averaged signal’s root-mean-square amplitude measured in standard deviations). All searches were conducted exclusively in the non-REM sleep stage, in accordance with literature suggesting a higher occurrence of sleep spindles during non-REM sleep, as well as higher functional relevance of non-REM sleep spindles^12,18,42^. The mean and standard deviation for non-REM sleep duration (and also other vigilance states) is reported in the Supplementary. Since sleep stage scoring and artefact removal were not independent for each electrode the values are equal for Fz and Cz within a dog and session.

### Statistical analyses

Independent samples t-tests were used to inquire if dogs of different sex and reproductive status were of significantly different age (in years). This was done to later exclude the possibility that age-effects are potentially explained by sex or reproductive status. Topographic differences in spindle features between Fz and Cz were tested using paired t-tests on the sub-sample of dogs (N = 84) that have data from both derivations. To test how spindle features (density, amplitude, frequency) might differ across age we used Generalized Linear Models (GLM) with robust model estimation, using age (in years) as a covariate, adding sex and reproductive status as fixed factors, and testing for the interactions sex × age and sex × reproductive status. The models were optimized with backwards elimination, excluding the least significant factors first (starting with interactions and keeping factors that are involved in significant interactions), until reaching the lowest absolute value for the Akaike criterion of model evaluation. The last factor removal was reversed if it resulted in a worse Akaike value and the final model is reported. Prior to testing, the residuals obtained for the initial model were examined for deviations from a normal distribution. If normality assumptions were violated (Kolmogorov-Smirnov test of normality P < 0.05) the distribution assumptions were adjusted to Gamma for spindle amplitudes and frequencies (recommended for variables with no possibility for negative values) and Tweedie for spindle density (recommended for variables with the possibility for zero values, but not negative values). Significant interactions between sex and reproductive status were followed by post-hoc tests comparing the effects of neutering for each sex, and sex differences in intact dogs (excluding dogs of unknown reproductive status). All analyses were repeated for the sub-sets of slow and fast spindles, separated as previously^18^ using the criterion applied in studies by Schabus and colleagues^19,55^ (fast spindles: spindles oscillating in a frequency ≥13 Hz, slow spindles: ≤13 Hz). We also repeated all analyses for detections in Fz and Cz. The need for outlier control analyses was determined visually (it appeared necessary in two analyses concerning amplitude) upon which outliers were identified based on standard scores (cases with a standard score above or below 2.68 for the variable in question were excluded). All analyses were performed with SPSS version 22.0.0.0.

## Results

### Control analyses

No age difference was found between male and female (t_153_ = 1.16, P = 0.248), nor between intact and neutered dogs (t_142_ = 1.548, P = 0.124).

The range, mean and standard deviations for density (spindles/minute), frequency (Hz) and amplitude (SD from baseline) for the total sample (across age, sex, reproductive status and breed) are provided in Table 1.

**Table 1:** Means and standard deviations for the density (spindles/minute), amplitude (measured in standard deviations from baseline) and frequency (in Hz) of detections in the sigma range (9–16 Hz) on midline electrodes Fz (frontal) and Cz (central), middle row is for Fz detections from subjects with an active Cz electrode.

recording channel	spindles/minute (M ± SD; range)	spindle amplitude (M ± SD; range)	spindle frequency (M ± SD; range)
Fz (N = 147)	3.6 ± 2.1; 0–11.7	1.9 ± 0.5; 1.3–4.2	10 ± 1.2; 8–16
fast spindles	0.5 ± 0.8; 0–4.7	2.1 ± 1.8; 1.1–17.3	13.9 ± 0.6; 13.1–16
slow spindles	3.3 ± 2; 0–11.7	1.8 ± 0.5; 1.2–4.2	9.5 ± 0.7; 7.8–11.7
Fz (N = 84)	4.1 ± 2.2; 0.6–11.7	1.8 ± 0.4; 1.4–3.6	10 ± 1.3; 8–16
fast spindles	0.6 ± 1; 0–4.7	1.5 ± 1.8; 1.1–12.6	11.2 ± 5.9; 13.2–16
slow spindles	3.7 ± 2; 0–11.7	1.8 ± 0.4; 1.4–3.6	9.5 ± 0.7; 8–11.7
Cz (N = 84)	4.1 ± 2.3; 0.2–9.7	1.9 ± 0.7; 1.4–7.2	10.9 ± 1.8; 7.9–15.6
fast spindles	1.3 ± 1.7; 0–7.9	2 ± 1; 1.2–8.5	14.2 ± 0.8; 13–16.6
slow spindles	3.2 ± 2; 0.2–9.3	1.9 ± 0.8; 1.4–7.7	9.9 ± 0.9; 7.7–12.3

### Topographic differences

Significantly more fast spindles were detected on Cz than Fz (53.6 ± 8.4 versus 20.5 ± 3.9, M ± SE; t_83_ = 4.314, P < 0.001) and a higher rate of spindles/minute (1.3 ± 0.2 versus 0.6 ± 0.1, M ± SE; t_83_ = 3.966, P < 0.001) was also observed. Furthermore fast spindles on Cz displayed a higher frequency (14.2 ± 0.1 versus 11.8 ± 0.6, M ± SE; t_76_ = 4.182, P < 0.001) and amplitude (2 ± 0.1 versus 1.6 ± 0.2, M ± SE; t_76_ = 2.472, P = 0.016).

No difference was found between Fz and Cz for the absolute occurrence of slow spindles (t_83_ = 1.056, P = 0.294), but density (spindles/minute) was higher on Fz (3.7 ± 0.2 versus 3.2 ± 0.2, M ± SE; t_83_ = 2.21, P = 0.03), slow spindle frequency was higher on Cz (10 ± 0.1 versus 9.5 ± 0.1, M ± SE; t_82_ = 4.568, P < 0.001). The amplitudes of slow spindles did not differ for detections on Fz and Cz (t_82_ = 1.415, P = 0.161).

**Figure 1:**
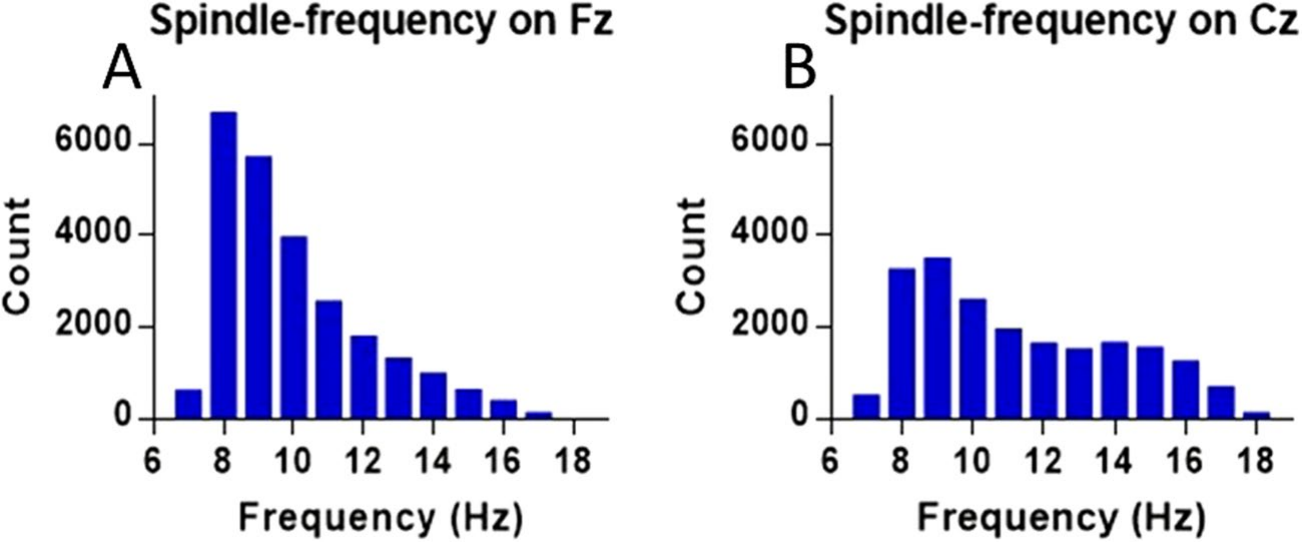
Number of detections for each frequency within the sigma range (9–16 Hz) found on Fz (N = 146, **A**) and Cz (N = 84, **B**).

The likelihood distribution for the frequency-content of all detections (across all dogs), on Fz and Cz are presented in Fig. 1.

For the GLM analyses we report only the significant findings. For models in which no predictor was significant see Supplementary Results. Figures summarize all findings relating to a particular spindling measure (density, amplitude, and frequency) across spindle subtypes and electrodes.

### Effects of age, sex and reproductive status on spindle density

For the subset of fast spindles the final model predicting spindle density on Fz included the factors age, sex, reproductive status and the interaction sex × reproductive status. Fast spindle density significantly increased with age (GLM, Wald Chi-Square = 8.107, P = 0.004) and there was a significant interaction between sex × reproductive status (GLM, Wald Chi-Square = 8.351, P = 0.004). Neutered males displayed a higher fast spindle density than intact males (0.5 ± 0.1 versus 0.2 ± 0.1, M ± SE; GLM, Wald Chi-Square = 5.793, P = 0.016), an opposite trend for more spindles/minute was observed in intact compared to neutered females (0.9 ± 0.3 versus 0.5 ± 0.1, M ± SE; GLM, Wald Chi-Square = 3.127, P = 0.077). Among intact dogs, females displayed a significantly higher density of fast spindles (0.9 ± 0.3 versus 0.2 ± 0.1, M ± SE; GLM, Wald Chi-Square = 12.827, P < 0.001), but there was no difference between male and female dogs among neutered animals (GLM, Wald Chi-Square = 0.061, P = 0.805).

For fast spindle density on Cz, the final model included the predictors sex, reproductive status and age, as well as the interactions sex × reproductive status and sex × age. However, among them only sex was significant (GLM, Wald Chi-Square = 5.588, P = 0.018), females displayed more fast spindles/minute than males (1.5 ± 0.3 versus 1.1 ± 0.2, M ± SE).

**Figure 2:**
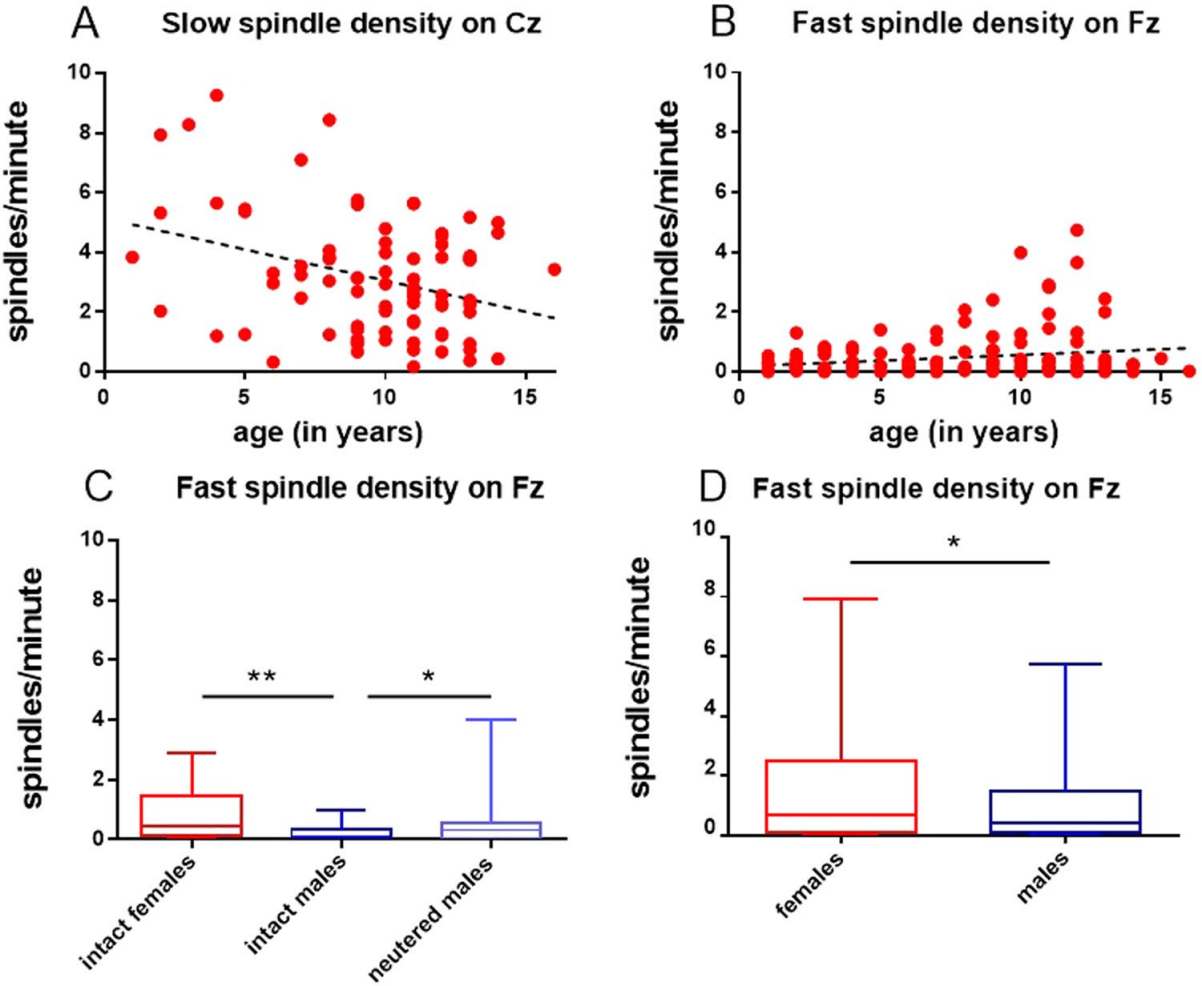
Spindle density as a function of age for slow spindles (≤13 Hz) on Cz (N = 84) (**A**), and fast spindles (≥13 Hz) on Fz (N = 147) (**B**). Boxplot graphs (minimum to maximum and interquartile distance) for fast (≥13 Hz) spindle density on Fz, intact females versus intact and neutered males (N = 79) (**C**); for fast (≥13 Hz) spindle density on Cz, female versus male dogs (N = 84) (**D**).

The final model for predicting slow spindle density on Cz included only age as a predictor. Slow spindle density declined with age (GLM, Wald Chi-Square = 7.4, P = 0.007). The results for density are summarized in Fig. 2.

### Effects of age, sex and reproductive status on spindle amplitude

The final model predicting the amplitude of fast (≥13 Hz) spindles on Fz included only sex as a predictor (GLM, Wald Chi-Square = 5.724, P < 0.017). Fast spindle amplitude was significantly higher in females compared to males (2.5 ± 0.3 versus 1.8 ± 0.1, M ± SE). The effect remained significant if outliers were removed (GLM, Wald Chi-Square = 5.034, P = 0.025).

The final model predicting the amplitude of slow (≤13 Hz) spindles on Fz included the factors age, sex and the interaction sex × age. Slow spindle amplitude declined with age (GLM, Wald Chi-Square = 4.169, P = 0.041).

**Figure 3:**
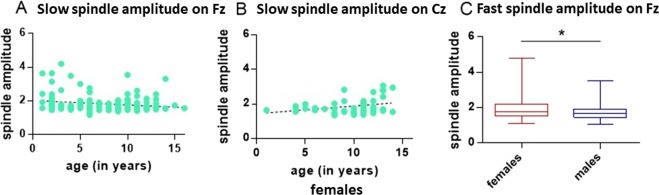
Slow spindle amplitude (≤13 Hz) on Fz (N = 145) as a function of age (**A**). Slow spindle amplitude, in females and on Cz, excluding one outlier (N = 45) (**B**). Boxplot graphs (minimum to maximum and interquartile distance) for fast (≥13 Hz) spindle amplitude, females versus males, for detections on Fz, excluding two outliers (N = 125) (**C**).

The final model predicting the amplitude of slow (≤13 Hz) spindles on Cz included the factors age, sex, and the interaction sex × age. The interaction sex × age was significant (GLM, Wald Chi-Square = 5.685, P = 0.017). Slow spindle amplitudes on Cz were significantly rising with age in females (GLM, Wald Chi-Square = 7.006, P = 0.008), but not in males (GLM, Wald Chi-Square = 0.109, P = 0.741). Excluding outliers, the age-related increase in amplitude for females remained significant (GLM, Wald Chi-Square = 6.818, P = 0.009). See Fig. 3 for a summary of these results.

### Effects of age, sex and reproductive status on spindle frequency

For fast (≥13 Hz) spindles on Fz the final model predicting frequency included the factors sex, reproductive status and the interaction sex × reproductive status. The interaction sex × reproductive status was significant (GLM, Wald Chi-Square = 4.014, P = 0.045). Intact females displayed higher fast spindle frequencies than neutered females (14.3 ± 0.1 versus 13.9 ± 0.1, M ± SE; GLM, Wald Chi-Square = 5.767, P = 0.016), but there was no difference between intact and neutered males (GLM, Wald Chi-Square = 0.385, P = 0.535). Among intact animals females displayed higher fast spindle frequencies than males (14.3 ± 0.1 versus 13.8 ± 0.2, M ± SE; GLM, Wald Chi-Square = 6.366, P = 0.012), but no difference was found between males and females in neutered animals (GLM, Wald Chi-Square = 0.098, P = 0.754).

For fast (≥13 Hz) spindle frequency on Cz the final model included the factors reproductive status and age. Fast spindle frequency was found to rise with age (GLM, Wald Chi-Square = 5.666, P = 0.017). Neutered animals displayed higher frequencies than intact animals (14.3 ± 0.1 versus 13.9 ± 0.1, M ± SE; GLM, Wald Chi-Square = 5.343, P = 0.021).

**Figure 4:**
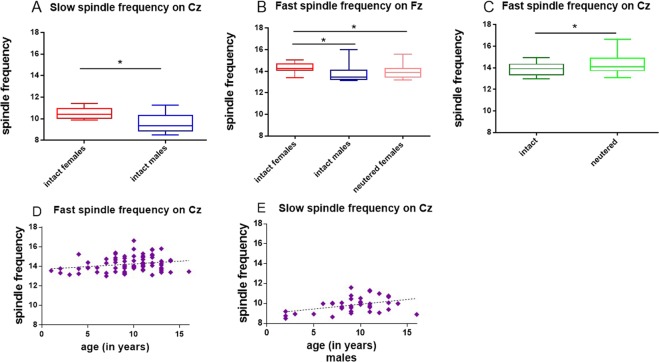
Spindle frequency among intact female and male dogs (N = 15) for slow (≤13 Hz) spindles on Cz (**A**); comparing intact females versus intact males and neutered females for fast spindles on Fz (≥13 Hz) (N = 80) (**B**); comparing all neutered against all intact animals, for fast spindles on Cz (N = 77) (**C**) boxplot graphs (minimum to maximum and interquartile distance). Fast spindle frequency for all dogs on Cz (N = 77) (**D**) and frequency of slow spindles in male dogs on Cz (N = 38) as a function of age (**E**).

For slow (≤13 Hz) spindle frequency on Cz the final model included the predictors age, sex, reproductive status and the interactions sex × age, sex × reproductive status. Slow spindle frequency was significantly predicted by the interaction sex × age (GLM, Wald Chi-Square = 6.023, P = 0.014) and sex × reproductive status (GLM, Wald Chi-Square = 4.406, P = 0.036). Slow spindle frequency on Cz did not change with age for females (GLM, Wald Chi-Square = 0.99, P = 0.32), but was rising in males (GLM, Wald Chi-Square = 7.262, P = 0.007). Among female dogs there was a trend for intact animals to display higher frequencies (10.5 ± 0.2 versus 10 ± 0.2, M ± SE; GLM, Wald Chi-Square = 3.113, P = 0.078) and no difference was found for intact versus neutered animals among males (GLM, Wald Chi-Square = 1.243, P = 0.265). Among intact animals females displayed higher frequencies than males (10.5 ± 0.2 versus 9.6 ± 0.3, M ± SE; GLM, Wald Chi-Square = 5.901, P = 0.015), no sex difference was observed in neutered dogs, however (GLM, Wald Chi-Square = 0.019, P = 0.89). These results are summarized in Fig. 4. Table 2 summarizes the significant findings from all models.

**Table 2:** Summary of all significant findings, sorted by significant predictors (age, sex, reproductive status), type of spindle (fast, slow), dependent measure (density, amplitude, frequency), and recording site (Fz, Cz).

Age-related associations			
type of spindle	characteristic	recording site	observation
fast	density	Fz	older > younger
fast	frequency	Cz	older > younger
slow	amplitude	Cz	older > younger (only in females)
slow	frequency	Cz	older > younger (only in males)
slow	density	Cz	older < younger
slow	amplitude	Fz	older < younger
**Sex-related associations**			
fast	density	Cz	females > males
fast	amplitude	Fz	females > males
fast	density	Fz	females > males (only in intact dogs)
fast	frequency	Fz	females > males (only in intact dogs)
slow	frequency	Cz	females > males (only in intact dogs)
**Reproductive status-related associations**			
fast	density	Fz	intact > neutered (only in females)
fast	frequency	Fz	intact > neutered (only in females)
slow	frequency	Cz	intact > neutered (only in females)
fast	density	Fz	neutered > intact (only in males)
fast	frequency	Cz	neutered > intact

## Discussion

After confirming that from several candidate definitions of canine sleep spindles, only automatic detections in the sigma range (9–16 Hz) predict post-sleep recall of novel information as in humans^18^, we turned to investigate spontaneously occurring spindles in a sample of 146 dogs with the same search criteria. Our aim was to test if the detection method, closely modelled on Nonclercq *et al*.^17^ can also reproduce relationships associated with aging/development and sexual dimorphism known from the human literature. Reproductive status was also investigated due to work directly linking spindling with the sexual hormones oestrogen and progesterone^45,46^. Given the modulating effects of sexual hormones on spindling features, neutering provides a proxy for pharmacological intervention, otherwise difficult to apply in non-invasive work with companion animal models. The dog literature is sparse on these questions. There is some marginal evidence suggesting that spindling might develop more slowly in the dog compared to other mammals^13,49^. Our previous findings also suggested a higher spindle density in female compared to male dogs^18^. The latter is in agreement with some human findings^22,44^, but not others^43,45^. A certain degree of homology between mammals can be assumed on the basis of the following findings: firstly, the involvement of thalamic structures, in particular the reticular thalamic nucleus in the generation of spindles/spindle-analogue events has been demonstrated for cats^32–34^, rodents^30,56–58^ and indirectly in humans^35^. Secondly, associations between spindles and cognitive performance, in particular learning, have been demonstrated in both humans^19,39–43,55^ and rodents^50,51,59^.

In humans^12,28,31^ and rodents^30^ a distinction between fast and slow spindles is not only accepted widely, but also based on topographic differences, with slow spindles (≤13 Hz) being localized predominantly in the frontal cortex and fast spindles (≥13 Hz) in central and parietal areas^28,30,31^. While previously in dogs we were able to obtain detections beyond 13 Hz, we did not observe a bimodal distribution for the detections’ frequency-content^18^, which is however reported in humans^31^. This was likely due to using a bipolar derivation with a signal recorded exclusively from a frontally placed electrode (Fz). Bipolar derivations tend to cancel out synchronous events happening far from the source and the frontal cortex is dominated by slow spindle activity^28^. In rats frontal fast spindles were not observed at all^30^. In the current sample, we had 86 dogs with an active Cz electrode and used this sub-sample to further verify if a distinction between slow and fast spindles is justified in the dog, by comparing the occurrence and features of both types between the detections made on Fz and Cz. As predicted by the topography outlined for humans^28^ fast spindles were more abundant, both in absolute numbers and as measured by density (spindles/minute) on Cz. In addition, we found central fast spindles to display higher amplitudes and frequencies. While this anterior-posterior gradient for fast spindles is the same as in humans^12^ and rats^30^, slow spindles were more abundant than fast spindles on both channels, which is more similar to what was observed in rats^30^. A likelihood distribution for the frequency content of spindling events approaches a bimodal distribution on Cz, but with a much flatter fast spindle peak. Interestingly, fast spindle frequency-content likelihood peaks at around 14 Hz, the average frequency of human fast spindles^12,28^, whereas slow spindles peak around 8–9 Hz, much lower than the suggested average frequency of human slow spindles at 12 Hz^12^.

Most developmental analogies to humans, like age-related decline in density^23^ and increase in (fast) spindle frequency^2^ were observed on Cz. The decline in density was more expressed for slow spindles, while frequency increased for fast spindles in all animals and for slow spindles in males. Age-related decline in spindle amplitudes (in our sample on Fz, in particular for slow spindles) in humans is specific to the transition from middle to old age and associated with cognitive decline^23,24,26,27^. Amplitude-associated findings are generally more reliable^45,54^. Computational modelling suggests that spindle amplitude increases with stronger feedforward inhibition of cortical pyramidal neurons by local interneurons^56^ in turn driven by thalamic inputs. Indeed, a loss of cortical interneurons and inhibitory synapses is part of the aging process^60^. Although this reduction is not exclusive to inhibitory cells and synapses^61^, such loss would directly limit the ability of the thalamus (where spindles begin^34^) to excite feedforward inhibition in the cortex (as predicted by the model of Sitnikova^56^).

Absolute ability to exclude alpha contamination in the measurement of slow spindles has been reasonably questioned, however, which might pose problems to slow spindle specific findings^12^. The overlap in defining frequency characteristics between slow spindles and alpha rhythms is in the 9–12 Hz range^12,62^. However, alpha activity in humans, recorded over central and parietal brain areas is increased in the elderly^63^, while evoked alpha measured over frontal areas increases from young to middle age^64^. Some authors have found no evidence of age correlations altogether^65^. It is therefore unlikely that our observations are explained by alpha activity, as slow spindle amplitude was found to drop with age over Fz, while no slow spindle specific findings were observed on Cz. Another concern for age-amplitude associations are anatomical differences, in particular between breeds and dogs of different sex with regards to skull thickness. The latter could influence the measurement of EEG amplitudes (see for instance Leissner *et al*.^66^). We do not rely, however, on absolute measures of amplitude^18^, moreover anatomical differences between female and male dogs have been argued minimal or absent^67^ in purebred dogs (they comprise 61.9% of our total sample). Our control analyses also show that the female and male part of the sample did not differ from each other with regard to age, neither did neutered and intact dogs, so that sex and age-related effects are unlikely confounded. Concerning breed-related differences (see Supplementary), the three largest breed-matched clusters in the data provided no evidence for breed-differences in amplitude.

We also report a set of humanlike sex differences in canine spindling. Higher amplitudes were observed in females on Fz. As with the link between spindle amplitude and age^23,24,26,27^ or general mental ability^54^, sex differences in amplitude are well replicated^43,45^ in humans, while the confounding effect of sexually dimorphic skull anatomy has been excluded^45^ and is not deemed a likely concern in (purebred) dogs^67^. Concerning spindle frequency, sexual differences in humans have more directly been linked to modulation by the sex hormones oestrogen and progesterone^45^. In humans higher fast spindle frequencies were observed in women, and in our study intact females also displayed higher fast spindle frequencies compared to both males and neutered females on Fz, while on Cz the frequency of slow spindles was also higher in intact female dogs. Interestingly, for fast spindles on Cz, frequency appeared to be generally higher for neutered animals, independent of sex. Since most of the literature on sleep spindles is derived from humans, we have little to speculate with about the general effects of neutering. The observed effects on females, however, allude more directly to the role of female sexual hormones like oestrogen and progesterone. The latter is a particularly important confirmation for the homology of human and dog spindles, as it suggests a similar “pharmacological” profile between the two oscillations. In the absence of invasive methods and in working with companion animals, neutering is a practically and ethically reasonable proxy to actual pharmacological interventions.

Concerning dog-specific findings, age and sex were associated with spindle density (spindles/minute) very similarly as in Iotchev *et al*.^18^. Density was rising with age and higher in females, although in the present sample both observations are specific to the fast variety (≥13 Hz). While in Iotchev *et al*.^18^ the age-related increase in spindles appeared more specific to the slow spindles, it should be noted that dogs were exposed to an experimental manipulation in which a higher slow spindle density was observed in the ‘learning condition’ compared to controls. The present results come from dogs that did not undergo any experimental procedures or handling prior to sleep and hence present ‘spontaneous’ activity patterns.

A higher spindle density was found for female dogs in Iotchev *et al*.^18^. In the present sample we see the same effect for the fast variety on both channels and more pronounced for intact dogs. In the human literature the issue is not settled, with some studies finding higher spindle occurrence in women^44^, but the opposite has been reported as well^43,45^. Part of the contradiction might be due to the menstrual cycle affecting spindle activity in women^45,46^, while some medical conditions, like depression, also appear to selectively increase spindle occurrence in female, but not male patients^21^. While the stage of the cycle was not being documented in the database from which our sample is derived, we find a higher variance in the density values of intact female dogs compared to intact male dogs (F tests for the equality of variances are reported in the Supplementary). This is to be expected in a sample (intact females) in which spindle expression is not stable over time and within an individual, and sampling was done randomly on females in different parts of their cycle. On Fz, neutering was observed to affect spindle density in males. So far, in humans, it is not settled whether density or amplitude differences between the sexes are more pronounced in interactions with age or with sexual hormones^45^, although an increase in spindle density in the high-progesterone luteal phase of the menstrual cycle has been described^46^. In the current study, we observed an age × sex interaction only with regards to amplitude on Cz, where spindle amplitudes were rising with age only in females, and in particular for slow spindles.

Overall, the similarity between the current study and Iotchev *et al*.^18^ concerning the effects of age and sex on spindle density in dogs, obtained with the same detection algorithm, supports the reliability of our measurement instrument for spindle detection in the dog.

Based on the reliability of our instrument and the accumulated analogies to human spindles from both studies, the evidence that we are really measuring sleep spindles in the dog is growing stronger. This is relevant to our dog-specific observation that the development of sleep spindles in the dog is not only delayed as suggested by previous work^49^, but also possibly slowed concerning frontal fast spindles. In humans, the rise and fall of spindle occurrence has also been tied specifically to the fast variety (oscillations ≥13 Hz)^19,27^ however, in humans the rising of fast spindle density is characteristic only of the transition childhood to adolescence^19,20^. Interestingly, this delayed or slowed development might be specific to frontal fast spindles, while slow and/or central spindles display a more expected pattern of linear amplitude and density decline across the lifespan of adult individuals^2,23,24,26^. However, an important additional difference is that in dogs the decline of amplitude and density are topographically dissociated.

Dogs of different weights and sizes have different life expectancies and it has been therefore suggested, that studies on aging in this species should account for this fact^68^. Previous efforts to study aging in dogs have seldomly adjusted age for breed, however^69,70^, instead, on some occasions circumventing the problem with one-breed samples^71^. We decided to use a heterogeneous dog population for the sake of generalizability. Since different cognitive functions are differently affected by dogs’ ageing^69,72^, and there is to our knowledge no theory in the human or rodent literature on how differences in life expectancy or size might affect the aging trajectory of spindles in particular, no correction factors have been used on subjects’ age in this study. Data with better breed representation will be needed to seriously tackle the question of how the ageing of sleep spindles varies in breeds with different life expectancies.

The mapping of where aging processes in dogs and humans converge and diverge is an important step in fully understanding dog’s potential as a model of cognitive aging, for which dogs have been argued a promising model species^3–5^. Our findings provide insight into the effects of aging on the canine sleeping brain. Importantly, we report evidence for a different course of aging with regard to fast and slow spindles in the dog. Generally, slow and central spindles appear to follow more similar trends to what has been observed in humans, while frontal fast spindles continue to increase in density. This latter development surprisingly mimics changes characterizing spindle development in humans that transition from childhood to adolescence^19,20^.

## Supplementary information

Supplementary

Dataset 1
