# Supplementary material for: Age-related differences and sexual dimorphism in canine sleep spindles
Source: Sci Rep. 2019 Jul 12;9:10092. doi: 10.1038/s41598-019-46434-y (PMC6626048; doi:10.1038/s41598-019-46434-y)
Supplement: Supplementary file 1 — Supplementary [file 41598_2019_46434_MOESM1_ESM.docx]

**Supplementary**

Age-related differences and sexual dimorphism in canine sleep spindles

Ivaylo Borislavov Iotchev^a^, Anna Kis^b^, Borbála Turcsán^a^, Daniel Rodrigo Tejeda Fernández de Lara^c^, Vivien Reicher^a^, Enikő Kubinyi^a^

^a^ Department of Ethology, Eötvös Loránd University, Budapest, Hungary

^b^ Institute of Cognitive Neuroscience and Psychology, Hungarian Academy of Sciences, Budapest, Hungary

^c^ Universidad Nacional Autónoma de México

**Methods**

*Electrode placement*

**
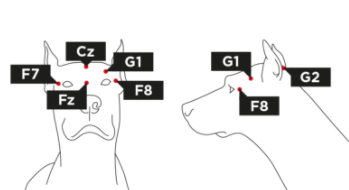
**

Supplementary Figure 1. Placement of the midline electrodes (Fz, Cz), eye-movement electrodes (F7, F8), ground (G1) and reference (G2) electrodes.^[[1]](#footnote-1)^

*Detection algorithm*

The algorithm was implemented in Matlab^®^. Detections were obtained from EEG segments marked as non-REM sleep (different stages of non-REM sleep are not clearly distinguishable in the dog). The EEG signal was filtered to remove electrical noise and artefacts. The maximal frequency of the stop band was 3 Hz, the high pass 5 Hz; low pass was set to 16 Hz, and the minimal frequency of the stop band to 35 Hz using a Butterworth filter with less than 0.5 dB ripple in the pass band and 30 dB attenuation. A second-order section representation of the Butterworth filter was applied for EEG data obtained with a sampling rate of 1024 Hz. The signal was then analysed with a Fast Fourier Transform (FFT) of 125 ms overlapping, Hanning-tapered 500 ms windows and zero-padded to support a 0.1 Hz resolution. The window length was set to the minimum duration of a spindle^6^ and as proposed in previous studies^7,53^. Except for the pre-filtering, all steps are modelled on propositions by Nonclercq et al.^53^. Our filter settings excluded more frequencies than proposed in the original description of the method^53^ in order to maintain a higher signal to noise ratio. The algorithm implements the following 3-step sequence:

1. The maximum power peak within a given segment was within the target range^53^ (9-16 Hz).
2. Amplitudes were calculated for each window as the root mean square (rms) of the corresponding signal^53^. Spindle amplitudes were previously proposed to be one standard deviation above the amplitude of the entire trace^6,53^, i.e. the filtered total of non-REM epochs^53^. Since EEG traces display high inter-individual variation in amplitude, standard scores (Z) were calculated for each window’s rms value relative to the population of rms values for a dog and session. If the standard score for a window was ≥ 1, the event was considered large enough to be a spindle.
3. To account for a subject’s individual range of amplitudes and frequencies, for each of these measures the true means and standard deviations for an individual dog and session were approximated using maximum likelihood estimation on the sample of detections obtained with step 1 and 2. An altered repetition of the first two criteria was then implemented in a final search: The frequency boundaries of the first criterion were redefined as ± 2 standard deviations from the estimated mean and the same was done for amplitude, using the estimated mean and standard deviation for amplitudes (using standard scores as in the 2^nd^ criterion and maintaining the 2^nd^ criterion for a minimum z-score ≥ 1). Events outside the adjusted range were discarded as outliers, but due to the altered criteria the final search could include events with frequencies outside the initial target range.

Time-windows whose content passed these criteria, were used to select from a corresponding array of time-points referencing the centre of each time-window. Time-points that were less than half a second apart^53^ were grouped as referring to the same spindle-event, then used to calculate the absolute number and density (spindles/minute). Average intra-spindle frequency was calculated across all time-windows that were marked as detections in the final step. Amplitudes were measured as the relative distance to the overall trace amplitude, using the standard scores calculated in step 2.

*Statistical Analyses*

To account for a possible impact of the recording settings on spindle-detection, we compared the density, amplitude and frequency of spindles on Fz between two groups defined by recording method (see methods), using independent samples t-tests. Detections on Cz were not compared, because all dogs with an active Cz channel used the same recording settings.

To account for possible artefacts caused by breed diversity in the sample, we also introduced a series of control tests, comparing the largest breed-matched sub-samples (≥ 8 dogs) with each other for differences in age, sex, reproductive status and spindle features (density, amplitude, frequency). ANOVA was applied for continuous dependent variables (age, spindle features), whereas breed-cohort differences in the ratios of the sexes or reproductive status were inquired with z-tests.

**Results**

*Descriptives*

Time spend awake (M ± SD; range): 73.5 ± 40.2; 11.3 – 181.7 (minutes)

Time in drowsiness (M ± SD; range): 42.5 ± 24.2; 0 – 122.7 (minutes)

Time in non-REM (M ± SD; range): 39.6 ± 26.8; 0 – 121.3 (minutes)

Time in REM (M ± SD; range): 21.7 ± 19.8; 0 – 80.7 (minutes)

*Control analyses*

For the full-sigma range (9-16 Hz) there was no difference in density (t_145_ = 1.064, P = 0.289), amplitude (t_144_ = 0.563, P = 0.574) or frequency (t_144_ = 0.041, P = 0.968) of the detections between dogs measured with different settings (see methods). For fast spindles (≥ 13 Hz) there was no difference in density (t_145_ = 0.704, P = 0.483), amplitude (t_125_ = 0.925, P = 0.357) or frequency (t_125_ = 0.182, P = 0.856) between recordings obtained with different settings. There was also no difference between dogs recorded with different settings regarding the density (t_145_ = 0.931, P = 0.354), amplitude (t_143_ = 0.705, P = 0.482) or frequency (t_143_ = 0.116, P = 0.908) of slow (≤ 13 Hz) spindles.

We tested for demographic differences (age, sex, reproductive status) in breed groups to exclude confounds between breed and demographics as potentially competing predictors of spindling features. Breed groups with at least 8 individuals were Border Collies (N = 14), Golden Retrievers (N = 9), and Hungarian Vizslas (N = 8). These breed groups did not differ significantly with regards to age (ANOVA, F_2_ = 1.635, P = 0.213), sex composition (z-test, P > 0.05) or reproductive status (z-test, P > 0.05).

We next tested if the largest breed-matched samples differed in spindling measures – density (spindles/minute), frequency and amplitude (for detections in the 9-16 Hz range). We found no difference in density (ANOVA, F_2_ = 1.776, P = 0.188) and amplitude (ANOVA, F_2_ = 0.057, P = 0.945), but there was a significant difference in frequency (ANOVA, F_2_ = 4.742, P = 0.017). Hungarian Vizslas displayed higher spindle frequencies than Border Collies (mean difference = 0.7, P = 0.01) and Golden Retrievers (mean difference = 0.8, P = 0.012).

*Final GLM models*

Final model for spindle density (full sigma-range, 9-16 Hz) on Fz

| predictors | Wald Chi-Square | P-values |
| --- | --- | --- |
| sex | 0.49 | 0.484 |
| age | 1.976 | 0.16 |
| reproductive satus | 1.298 | 0.255 |
| sex x reproductive status | 3.076 | 0.079 |

Final model for spindle density (full sigma-range, 9-16 Hz) on Cz

| predictors | Wald Chi-Square | P-values |
| --- | --- | --- |
| **age** | **4.940** | **0.026** |

Final model for fast (≥ 13 Hz) spindle density on Fz

| predictors | Wald Chi-Square | P-values |
| --- | --- | --- |
| **sex** | **6.614** | **0.01** |
| **age** | **8.107** | **0.004** |
| reproductive status | 0.002 | 0.964 |
| **sex x reproductive status** | **8.351** | **0.004** |

Final model for fast (≥ 13 Hz) spindle density on Cz

| predictors | Wald Chi-Square | P-values |
| --- | --- | --- |
| **sex** | **5.588** | **0.018** |
| age | 1.425 | 0.233 |
| reproductive status | 0.533 | 0.465 |
| sex x age | 2.515 | 0.113 |
| sex x reproductive status | 2.294 | 0.130 |

Final model for slow (≤ 13 Hz) spindle density on Fz

| predictors | Wald Chi-Square | P-values |
| --- | --- | --- |
| **reproductive status** | **3.855** | **0.05** |

Final model for slow (≤ 13 Hz) spindle density on Cz

| predictors | Wald Chi-Square | P-values |
| --- | --- | --- |
| **age** | **7.4** | **0.007** |

Final model for spindle amplitude (full sigma-range, 9-16 Hz) on Fz

| predictors | Wald Chi-Square | P-values |
| --- | --- | --- |
| sex | 0.389 | 0.533 |
| **age** | **4.117** | **0.042** |
| sex x age | 2.144 | 0.143 |

Final model for spindle amplitude (full sigma-range, 9-16 Hz) on Cz

| predictors | Wald Chi-Square | P-values |
| --- | --- | --- |
| sex | 2.652 | 0.103 |
| **age** | **4.351** | **0.037** |
| **sex x age** | **4.998** | **0.025** |

Final model for fast (≥ 13 Hz) spindle amplitude on Fz

| predictors | Wald Chi-Square | P-values |
| --- | --- | --- |
| **sex** | **5.724** | **0.017** |

Final model for fast (≥ 13 Hz) spindle amplitude on Cz

| predictors | Wald Chi-Square | P-values |
| --- | --- | --- |
| sex | 0.191 | 0.662 |
| reproductive status | 0.618 | 0.432 |
| sex x reproductive status | 2.877 | 0.09 |

Final model for slow (≤ 13 Hz) spindle amplitude on Fz

| predictors | Wald Chi-Square | P-values |
| --- | --- | --- |
| sex | 0.397 | 0.528 |
| **age** | **4.169** | **0.041** |
| sex x age | 1.594 | 0.207 |

Final model for slow (≤ 13 Hz) spindle amplitude on Cz

| predictors | Wald Chi-Square | P-values |
| --- | --- | --- |
| age | 4.07 | 0.044 |
| sex | 3.6 | 0.058 |
| **sex x age** | **5.685** | **0.017** |

Final model for spindle frequency (full sigma-range, 9-16 Hz) on Fz

| predictors | Wald Chi-Square | P-values |
| --- | --- | --- |
| reproductive status | 1.606 | 0.205 |

Final model for spindle frequency (full sigma-range, 9-16 Hz) on Cz

| predictors | Wald Chi-Square | P-values |
| --- | --- | --- |
| **sex** | **8.594** | **0.003** |
| age | 1.705 | 0.192 |
| reproductive status | 0.009 | 0.923 |
| sex x age | **4.252** | **0.039** |
| sex x reproductive status | **5.615** | **0.018** |

Final model for fast (≥ 13 Hz) spindle frequency on Fz

| predictors | Wald Chi-Square | P-values |
| --- | --- | --- |
| **sex** | **5.394** | **0.02** |
| reproductive satus | 1.085 | 0.298 |
| **sex x reproductive status** | **4.014** | **0.045** |

Final model for fast (≥ 13 Hz) spindle frequency on Cz

| predictors | Wald Chi-Square | P-values |
| --- | --- | --- |
| **age** | **5.666** | **0.017** |
| **reproductive status** | **5.343** | **0.021** |

Final model for slow (≤ 13 Hz) spindle frequency on Fz

| predictors | Wald Chi-Square | P-values |
| --- | --- | --- |
| sex | 1.682 | 0.195 |

Final model for slow (≤ 13 Hz) spindle frequency on Cz

| predictors | Wald Chi-Square | P-values |
| --- | --- | --- |
| **sex** | **9.852** | **0.002** |
| age | 0.457 | 0.499 |
| reproductive status | 0.393 | 0.531 |
| **sex x age** | **6.023** | **0.014** |
| **sex x reproductive status** | **4.406** | **0.036** |

*Differences in variance*

The tests of equal variance available in GraphPad Prism were used to compare variance differences between male and female dogs, as well as neutered and intact animals in our post-hoc tests. The variance for fast spindle density on Fz was greater for neutered than intact male dogs (F = 7.719, P < 0.001). Among intact animals, females showed a greater variance for fast spindle density on Fz than males (F = 13.37, P < 0.001). There was no difference in fast spindle density variance between female and male dogs on Cz (F = 1.493, P = 0.2123).

Fast spindle amplitude variance was greater for females than males on Fz (F = 27.35, P < 0.001).

Spindle frequency variance on Cz was not different between intact males and females, but there was a trend for males to display a greater variance (F = 6.158, P = 0.0956). There was no difference between intact female and male dogs’ variance of slow spindle frequency (F = 2.587, P = 0.3735). The variance of fast spindle frequency on Fz was also not different between intact females and intact males (F = 2.394, P = 0.1604), nor between intact and neutered females (F = 1.489, P = 0.5103) for detections on Cz from the full sigma-range. The variance of neutered females compared to intact females was, however, higher for fast spindle frequency on Cz (F = 9.891, P = 0.0371).

1. Figure by Vivien Reicher, co-author of this publication [↑](#footnote-ref-1)
